# Opening new frontiers with catalytic nucleic acids in miRNA inhibition

**DOI:** 10.3389/fphar.2025.1604711

**Published:** 2025-06-23

**Authors:** Olga Patutina, Svetlana Miroshnichenko, Daria Chiglintseva, Marina Zenkova

**Affiliations:** Laboratory of Nucleic Acids Biochemistry, Institute of Chemical Biology and Fundamental Medicine SB RAS, Novosibirsk, Russia

**Keywords:** catalytic nucleic acids, miRNA, antimiRs, DNAzyme, ribozyme, antimiRzymes, artificial ribonuclease, miRNase

## Abstract

The concept of utilizing synthetic nucleic acids and their conjugates with biologically active molecules as RNA-targeted therapeutic agents represents a powerful strategy in the treatment of human pathologies. Recent research demonstrates that neoplastic development is closely associated with dysregulation of miRNAs, which are essential regulators of gene expression, highlighting the potential of therapeutic strategies aimed at their inhibition. Current approaches to pathological microRNA (miRNA) regulation primarily rely on physical blocking or sequestration mechanisms. However, these non-enzymatic strategies are limited by their stoichiometric nature, necessitating high drug doses to achieve therapeutic efficacy. A promising alternative lies in the application of catalytic nucleic acids, including miRNA-targeted ribozymes, DNAzymes/XNAzymes (antimiRzymes), and artificial ribonucleases (miRNases), which enable selective suppression of overexpressed miRNAs in pathological conditions through multiple enzymatic cleavage events. This review examines the fundamental principles governing the design of currently developed antimiRzymes and miRNases, analyzes their ribonuclease activity using synthetic miRNA substrates, and discusses key achievements in miRNA-inhibiting capability in tumor cells, along with their antitumor effects. Being effective RNA cleavers, these catalytic nucleic acids demonstrate remarkable potential, often surpassing the efficacy of conventional antisense oligonucleotides, and represent a promising therapeutic modality for RNA-associated diseases.

## 1 Introduction

The development of RNA-targeting drugs represents a breakthrough direction of research in molecular therapeutics, enabling specific genetic regulation and offering novel treatment approaches for challenging biological targets that were previously unresponsive to conventional pharmaceuticals. This therapeutic approach is particularly valuable given the vast number of disease-associated RNAs, including coding and non-coding RNAs, identified through genomic and transcriptomic studies, and offering numerous potential targets for drug development. In recent years, there has been growing interest in targeting non-coding RNAs, including long non-coding RNAs, microRNAs, circRNAs, piRNAs and tRFs (Chen et al., 2021; Chen et al., 2025; Di Fazio and Gullerova, 2023; Fu et al., 2023; Mattick et al., 2023; Nasseri et al., 2023; Aquino-Jarquin, 2024; Jiang et al., 2024). Historically categorized as non-functional genomic elements, these molecules are now recognized as pivotal regulators of gene expression and disease pathogenesis.

MicroRNAs (miRNAs) are one of the most studied types of non-coding RNAs, which have been shown to play an overriding role in the gene expression regulation network, coordinating a diverse array of vital biological processes (O’Brien et al., 2018; Dexheimer and Cochella, 2020; Bofill-De Ros and Vang Ørom, 2024). The extensive involvement of miRNAs in cellular maintenance and functionality, along with their dramatic dysregulation under pathological conditions, positions them as promising therapeutic targets. For instance, the critical roles of miRNAs in regulating myocardial cell morphology, vascular smooth muscle differentiation, and neovascularization establish them as essential targets in cardiovascular diseases (Vishnoi and Rani, 2023). Similarly, their contribution to reprogramming fibroblasts into neurons, and modulating epigenetic modifications underscores their pivotal role in neurodevelopmental and psychiatric disorders (Colită et al., 2024; Sun et al., 2024). Furthermore, their multifaceted influence on proliferation, migration, apoptosis, and invasion solidifies their status as key drivers of tumorigenesis. Altered miRNA expression frequently leads to aberrant signaling pathways and the upregulation of oncogenic factors, thereby accelerating aggressive tumor progression (Rupaimoole and Slack, 2017; Gebert and MacRae, 2019; Inoue and Inazawa, 2021). The strategic suppression of miRNA represents an emerging therapeutic modality with significant potential in various disease treatments including cancer (Miroshnichenko and Patutina, 2019; Miroshnichenko et al., 2019b; Lee et al., 2020; Fu et al., 2021; Gaponova et al., 2022; Hussen et al., 2023; Chiglintseva et al., 2024; Seyhan, 2024).

## 2 MiRNA: biogenesis and functional dynamics

Given the critical role of miRNAs in various biological processes, the mechanisms underlying their biogenesis and functioning have been extensively studied. There is a main canonical pathway of miRNA maturation and more specific non-canonical pathways (Bartel, 2018; Michlewski and Cáceres, 2019). In brief, in the canonical biogenesis pathway, the intricate step-by-step process of miRNA generation involves the transcription of miRNA-coding sequences (localized as individual “miRNA genes,” in introns or in gene untranslated regions) by polymerase II, sequential catalytic processing of precursor pri-miRNA by Drosha/DGCR8 (Microprocessor complex) to pre-miRNA and finally by Dicer to 22-nucleotide duplex. Subsequently, the generated duplex is loaded into Argonaute protein (Ago 1–4) resulting in maturation of the mature guide strand of the duplex by degradation of the passenger strand with a final assembly of miRNA-induced silencing complex (miRISC) responsible for induction of post-transcriptional gene regulation. Comprehensive details regarding the synthesis and maturation of miRNAs can be found in the following reviews (Komatsu et al., 2023; Shang et al., 2023). While most miRNAs are formed through the canonical biogenesis pathway, described above, there are alternative pathways in which some processing steps are skipped. These alternative pathways include the Dicer-independent pathway, the Drosha/DGCR8-independent pathway (mirtrons), and a pathway in which both processing steps are absent (agotrons) (Ruby et al., 2007; Babiarz et al., 2008; Yang et al., 2010; Hansen et al., 2016). Agotrons are specific sequences that, after transcription, undergo splicing in the cell nucleus, then are transported to the cytoplasm and bind directly to Ago proteins to regulate gene expression (Hansen et al., 2016). All biogenesis pathways culminate in the formation of mature miRNA molecules and the subsequent assembly of the miRISC complex.

**FIGURE 1:**
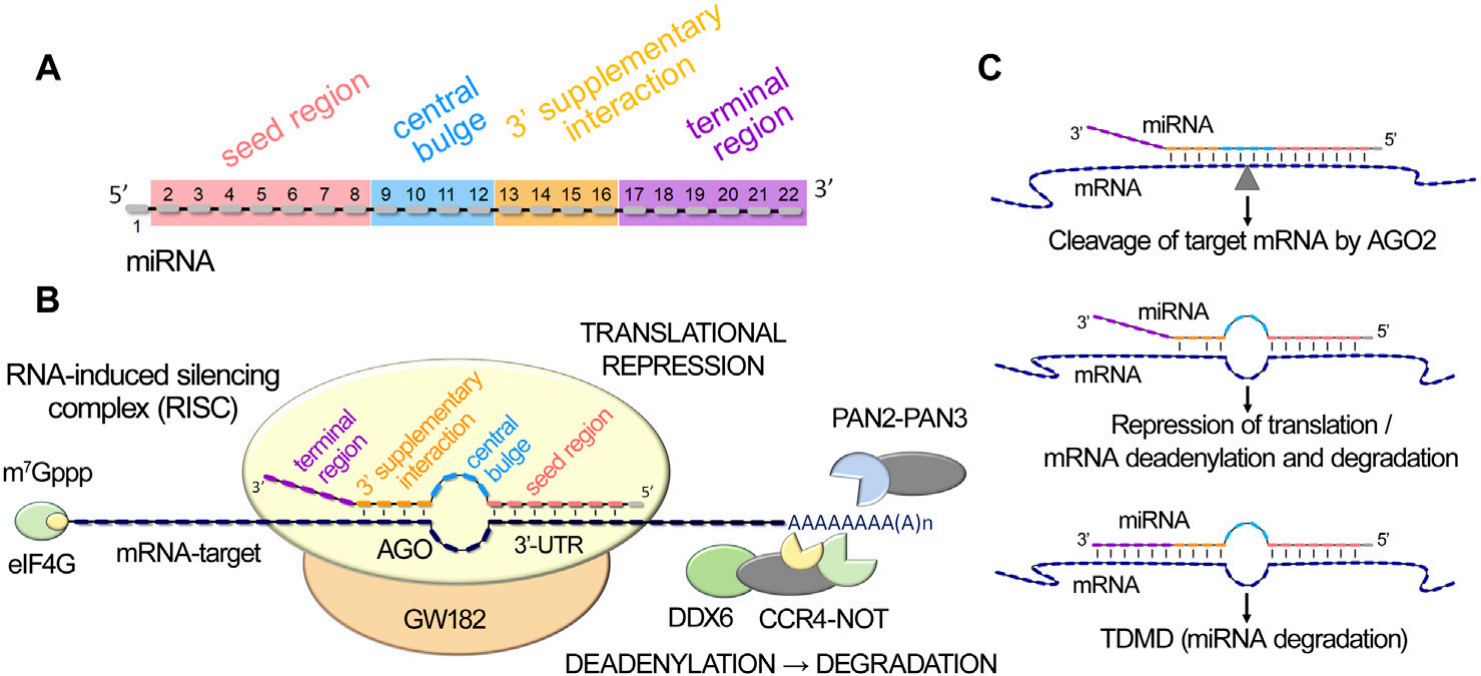
MiRNA-mediated post-transcriptional gene silencing. **(A)**. MiRNA functional domains: seed region, central bulge, 3′-supplementary interaction, and terminal region. **(B)**. Schematic illustration of miRISC (miRNA-induced silencing complex) interaction with target mRNA, including miRNA binding to mRNA 3′-UTR through seed and 3′-supplementary interaction regions, subsequent engagement of DDX6 helicase and the PAN2-PAN3 and CCR4-NOT deadenylation complexes, leading to translational repression, mRNA destabilization and degradation. **(C)**. Schematic representation of miRNA duplex variants, the formation of which leads to the initiation of AGO2-mediated cleavage of the target mRNA, suppression of translation, or destabilization and degradation of mRNA, or the initiation of miRNA decay process - TDMD (Target-Directed MicroRNA Decay).

miRISC interacts with target mRNAs to mediate post-transcriptional gene silencing through the initial complementary binding of miRNA with the 3′-untranslated region of mRNA. Recent studies have identified four key functional elements in miRNA sequence that ensure their biological activity through coordinated step-by-step functioning (Grimson et al., 2007; Becker et al., 2019) (Figure 1A). The seed region (2–7 nts) and the subsequent pairing in the 3′-supplementary interaction region (13–16 nts) play a crucial role in mediating miRNA-to-mRNA targeting (Figure 1A). Once such a complex is formed, the central loop (8–12 nts) and the terminal region (17–21/25 nts) of miRNA facilitate its conformational reconstruction, inducing effective repression of mRNA targets (Bartel, 2018; Becker et al., 2019; Pawlica et al., 2019; Willkomm et al., 2022; Mohamed et al., 2025) (Figures 1A,B).

In the well-established canonical pathway of miRNA function, the interaction of miRISC with target mRNA can lead to three main post-transcriptional regulatory events: (1) endonucleolytic cleavage of the target mRNA by AGO2, (2) translational repression, and (3) deadenylation, followed by subsequent mRNA degradation (Willkomm et al., 2022) (Figure 1C). The primary requirement for AGO2-mediated mRNA cleavage is base pairing simultaneously in the seed, central and supplementary interaction regions. Formation of a duplex exclusively in the seed region, as well as the establishment of additional complementary pairs in the supplementary interaction domain, initiates cleavage-independent AGO2-mediated regulation, including repression of translation and mRNA degradation (Willkomm et al., 2022) (Figure 1C). miRNA-activated repression of translation and mRNA destabilization are mediated by the TNRC6 (GW182 family) repression machinery, which engages DDX6 helicase for translational repression and recruits the PAN2-PAN3 and CCR4-NOT deadenylation complexes (Mauro et al., 2023; Pavanello et al., 2023), leading to poly(A) tail shortening (Figure 1B). In mammals, mRNA decay, including translational repression, destabilization with subsequent degradation, without engaging AGO-2-mediated endonucleolytic mRNA cleavage, is predominant (Bartel, 2018). Notably, extensive base pairing with the 3′-terminal region of a miRNA, in conjunction with mismatches in the central region, triggers a process of intrinsic miRNA utilization called Target-Directed MicroRNA Decay (TDMD), leading to the degradation of the miRNA itself as a part of miRNA turnover in cells (Fuchs Wightman et al., 2018; Willkomm et al., 2022; Han and Mendell, 2023; Shang et al., 2023) (Figure 1C).

In addition to its canonical role in post-transcriptional gene regulation, increasing evidence is emerging about the broader functions of miRNAs. miRNAs are important regulators at multiple levels, performing their functions not only in the cytoplasm but also in the mitochondria and nucleus. Nuclear miRNAs exhibit multiple regulatory functions, primarily controlling both coding and non-coding RNA transcription (Liu H. et al., 2018; Hu et al., 2023; Billi et al., 2024; Diener et al., 2024). These molecules can regulate pri-miRNA maturation and long non-coding RNA levels, while also participating in chromatin remodeling and alternative splicing regulation. When localized in the nucleolus, miRNAs interact with ribosomal RNAs, influencing their biogenesis and repression. Furthermore, nuclear miRNAs can mediate transcriptional gene activation (TGA) and silencing (TGS) through direct interaction with promoter regions and recruitment of regulatory protein complexes that modify chromatin structure.

## 3 The role of miRNAs in oncogenesis

Recent studies have demonstrated that dysregulated miRNA expression patterns play a fundamental role in tumor development (Wu et al., 2024). The perturbation of miRNA expression during neoplastic transformation encompasses multiple molecular mechanisms, including genomic alterations, such as deletions and amplifications of miRNA loci, genetic mutations affecting miRNA-encoding sequences, modifications to epigenetic and transcriptional regulatory networks including those mediated by methyltransferases, RNA binding proteins and DNA repair proteins, which lead to post-transcriptional processing alterations, and disruptions in the miRNA biogenesis, maturation and sorting (Hata and Lieberman, 2015; Annese et al., 2020; Urbanek-Trzeciak et al., 2020; Pajares et al., 2021; Mangiapane et al., 2024). As a result, malignant transformation is characterized by distinctive alterations in miRNA profiles, manifesting as either upregulation or downregulation of specific miRNAs relative to their expression in normal tissue (Zhang et al., 2007). The pathogenic accumulation of oncogenic miRNAs facilitates neoplastic progression through the suppression of tumor-suppressor genes, while the systemic depletion of tumor-suppressive miRNAs results in oncogene overexpression. It is noteworthy, however, that this functional categorization exhibits contextual character, as certain miRNAs may demonstrate opposing functions in different neoplastic contexts (Svoronos et al., 2016).

Alterations in specific miRNA expression levels have demonstrated robust correlations with tumor progression, metastatic potential, drug resistance and adverse survival outcomes in oncological patients (Si et al., 2019; Sempere et al., 2021; Smolarz et al., 2022). As a result, miRNAs are now considered valuable diagnostic and prognostic markers. Among the most extensively characterized oncogenic miRNAs are miR-221/222, miR-155, miR-21, miR-10b (Hata and Lieberman, 2015; Sheedy and Medarova, 2018; Bautista-Sánchez et al., 2020; Hussen et al., 2025), whereas the tumor-suppressive category prominently includes members of the let-7 family, miR-15a/16-1, miR-200 (Hata and Lieberman, 2015; Sewastianik et al., 2021).

The intricate nature of miRNA-mediated regulation is additionally underscored by their pleiotropic effects, as individual miRNAs can modulate multiple target genes and orchestrate diverse signaling cascades, highlighting their potential as promising therapeutic targets in cancer treatment.

## 4 Cutting-edge technologies for suppressing dysregulated miRNAs

Strategies aimed at restoring or inhibiting specific disease-associated miRNAs in a sequence-specific manner offer innovative approaches for targeted therapies. To date, a number of advanced technologies have been developed to effectively reduce the excessive activity of miRNAs. The CRISPR/Cas9 system has proven successful in reducing the abundance of mature miRNA molecules through precise genome editing near precursor miRNA loci (Chang et al., 2016; Yang et al., 2018; Hussen et al., 2023). As an alternative strategy, mRNA target protectors have been introduced, utilizing miRNA-masking oligonucleotides to impede the interaction between miRNAs and their target mRNAs by binding to their 3′-untranslated regions (Choi et al., 2007; Wang, 2011). Additionally, various oligonucleotide-based loss-of-function strategies have been developed, including: anti-miRNA oligonucleotides that directly knock down target miRNAs by complementary pairing (Boutla, 2003; Zhang et al., 2016; Lennox et al., 2017; Lima et al., 2018; Zeniya et al., 2018; Miroshnichenko et al., 2019b; Patutina O. A. et al., 2020; Gaponova et al., 2022), miRNA sponges consisting of multimeric oligonucleotides with miRNA binding sites (Ebert et al., 2007; Ebert and Sharp, 2010; Liu et al., 2014; Gao et al., 2015; Liang et al., 2016; Mignacca et al., 2016; Jie et al., 2022), and synthetic circular RNAs (circRNAs) serving as competitive endogenous RNAs (ceRNAs) to sequester miRNAs (Liu X. et al., 2018; Müller et al., 2020; Rama et al., 2022). Some current research focuses on developing multicomponent nanomachines based on bifunctional DNAzymes that simultaneously inhibit miRNAs through binding while cleaving the target gene mRNA (Wang et al., 2022; Yan et al., 2023a). Moreover, there has been a growing interest in the synergistic application of miRNA-based approaches alongside conventional drug treatments, which also holds significant promise in the field of therapeutic interventions (Kasinski et al., 2015; Chaudhary et al., 2017; Miroshnichenko and Patutina, 2019).

Although antisense-mediated technologies have certain strategy-specific challenges, they represent a promising approach for targeted RNA modulation. Currently, several oligonucleotide-based therapeutics intended to inactivate disease-associated miRNAs are undergoing or have completed clinical trials. Notably, in 2023, the successful completion of phase 1 clinical trials for LNA-i-miR-221 was reported. This 13-mer phosphorothioate (PS) and locked nucleic acids (LNA)-modified oligonucleotide targeting the oncogenic miR-221 was applied for the treatment of refractory multiple myeloma (ClinicalTrials.gov ID: NCT04811898) (Tassone et al., 2023). Additionally, FDA-approved drug Cobomarsen (MRG-106), designed to combat the highly oncogenic miR-155, has been integrated into clinical practice as a therapy for various malignant blood diseases, including cutaneous T-cell lymphoma (ClinicalTrials.gov ID: NCT03837457) (Tassone et al., 2023). The successful completion of Phase 1 and Phase 2 clinical trials has been reported for the anti-miR-17 therapeutic RGLS4326 and the anti-miR-21 agent RG012, respectively. These drugs are designed to address distinct forms of kidney diseases, including autosomal dominant polycystic kidney disease and Alport syndrome (ClinicalTrials.gov IDs: NCT04536688, NCT03373786) (Gomez et al., 2015; Kashtan and Gross, 2021). Additionally, a novel LNA-modified antisense oligonucleotide designed to inhibit miR-132 for treatment of heart failure has successfully completed Phase 1 clinical trials (ClinicalTrials.gov ID: NCT04045405) (Batkai et al., 2021; Täubel et al., 2021). Furthermore, the anti-miR-103/107 therapeutic RG-125/AZD4076 is actively undergoing Phase 1 clinical trials. This study focuses on evaluating the safety and tolerability of the drug in patients with non-alcoholic steatohepatitis (ClinicalTrials.gov IDs: NCT02612662, NCT02826525) (Huang, 2017; Drenth and Schattenberg, 2020). Such progress highlights the significance and promising potential of miRNA-based therapeutics.

## 5 A new frontier: catalytic nucleic acids as miRNA-specific inhibitors

Efficient inhibition of miRNAs represents significant challenges due to the elevated production rates (e.g., for miR-21 ∼ 130 copies/cell/min), high concentrations (form 1,000 to 30,000 copy numbers compared to mRNAs typically accounting for fewer than 100 per cell), and prolonged life-time (25 h *versus* 2.2 h for mRNAs) (Ragan et al., 2011; Kingston and Bartel, 2019; Zlotorynski, 2019). Powerful approaches are needed for rapid, irreversible inactivation of emerging miRNA targets, especially during pathogenesis.

Current strategies for modulating disease-associated miRNAs mainly involve physical blocking or sequestering their activity. These non-enzymatic approaches have limited turnover capability, as they can neutralize only a finite number of miRNA molecules, necessitating stoichiometric doses to achieve a significant effect. Alternative advanced approaches to selectively inhibit overexpressed miRNAs in a pathological context involve the utilization of catalytic nucleic acids, such as miRNA-targeted ribozymes, DNAzymes/XNAzymes (antimiRzymes) or artificial ribonucleases (miRNases), which are capable of providing cleavage of miRNA in an enzymatic-like multiple turnover manner.

**FIGURE 2:**
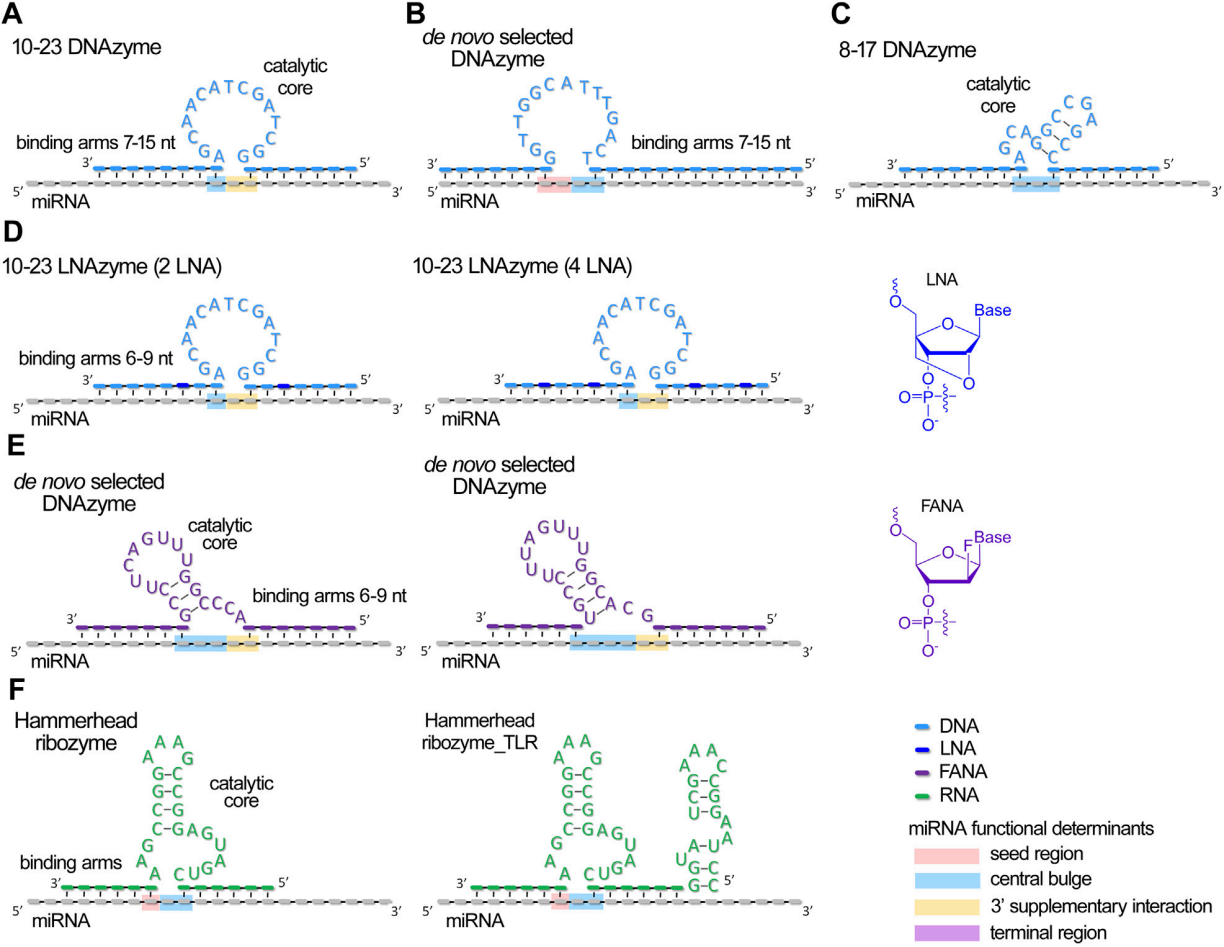
Schematic structures of currently developed antimiRzymes (miRNA-targeted DNAzymes and ribozymes) and their complexes with miRNA. **(A–E)** Structures of DNAzymes targeted to miRNAs. **(F)**. Structures of ribozymes targeted to miRNAs. miRNA functional domains targeted by catalytic core of antimiRzymes are highlighted by colors: pink – seed region, blue – central bulge, orange – 3′ supplementary region, and purple – terminal region.

A ribozyme or DNAzyme is a single-stranded RNA or DNA motif with autocatalytic cleavage activity, featuring a catalytic core flanked by two variable arms that facilitate complementary binding to the target RNA molecule (Figure 2). Ribozymes and DNAzymes have shown great potential in diverse advanced applications, including disease biomarker detection, intelligent release systems, therapeutic mRNA suppression, and biosensors for metal ion detection, as well as controlling materials assembly and driving nanoscale machines (Liu et al., 2009; Gong et al., 2015; Peng et al., 2017; Zhou et al., 2017; Ma and Liu, 2020; Micura and Höbartner, 2020; Yin et al., 2020; Wang Y. et al., 2021; Li F. et al., 2022; Taylor et al., 2022). Due to their ability to target virtually any RNA, these catalytic nucleic acids can be designed to specifically cleave and suppress miRNAs, offering new therapeutic possibilities.

**FIGURE 3:**
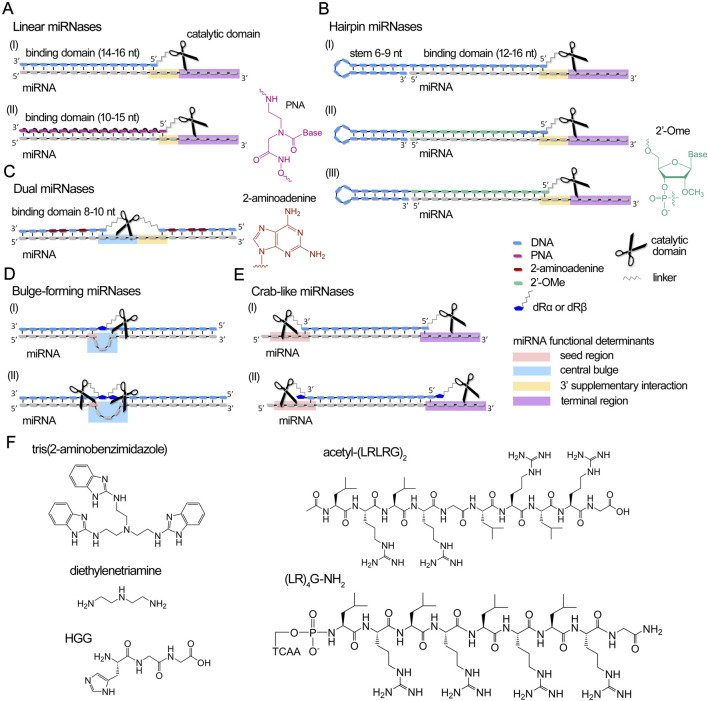
Design concept of currently developed miRNases (miRNA-targeted artificial ribonucleases) and their complexes with miRNA. **(A–E)** Different structural design of recognition motifs of miRNases, including linear **(A)**, hairpin **(B)**, dual **(C)**, bulge-forming **(D)** and crab-like **(E)** variants. miRNA domains targeted by miRNases are highlighted by colors: pink – seed region, blue – central bulge, orange – 3′ supplementary region, and purple – terminal region. dRα and dRβ – abasic sugar residue with aminohexyl linkers attached to an anomeric C1′-carbon either in α- or β-orientation. **(F)**. Chemical structures of catalytic domains of miRNases.

A miRNase is a hybrid molecule that combines an oligonucleotide domain for target RNA binding and a covalently attached catalytic group, such as imidazole (Danneberg et al., 2015; Zellmann et al., 2019; 2024), amine derivatives (Gaglione et al., 2011) or short peptides (Gaglione et al., 2011; Patutina et al., 2017a; Patutina et al., 2018), capable of catalyzing the cleavage of the RNA substrate (Figure 3). A detailed overview of the design, synthesis, and application of aRNases is covered in the following review (Staroseletz et al., 2021). This approach can be effectively utilized for the therapeutic multi-turnover degradation of dysregulated miRNAs. For biological applications, aRNases that utilize a catalytic peptide as the cleavage domain have demonstrated considerable success (Patutina et al., 2019; Chiglintseva et al., 2024).

Unlike prevailing miRNA-blocking or sequestering methods, due to their catalytic mode of action, catalytic nucleic acids-based compounds are capable of directly cleaving target RNA molecules, leading to irreversible inactivation of pathogenic miRNAs. Unlike canonical antisense oligonucleotides, XNAzymes and artificial ribonucleases function as autonomous molecular agents, operating independently without the need to engage the intracellular RNA silencing machinery.

Depending on the structural configuration of ribozymes/XNAzymes and artificial ribonucleases, the focus of nucleolytic activity can be directed towards specific miRNA functional determinants (Figure 1B). Although all four functional segments of miRNA (*viz*. seed region, central loop, 3′-supplementary interaction, terminal region) are involved in post-transcriptional mRNA repression, the seed region is recognized as the primary factor ensuring targeting specificity and efficiency and is the main site for silencing by developed miRNA-specific blocking oligonucleotides (Obad et al., 2011; Staedel et al., 2015).

Since the catalytic mechanism involves destruction of specific miRNA motifs, resulting in the complete loss of their coordinated function, catalytic oligonucleotides can be designed to target any critical region within the miRNA molecule, which undoubtedly impairs miRNA activity. Various structures of antimiRzymes and aRNases have been designed to target different functional segments of miRNA for catalytic attack (Figures 2, 3). A more thorough analysis of this issue will be presented in the section below.

## 6 Major determinants of catalytic activity of antimiRzymes and miRNases

Various structural variants of antimiRzymes and miRNases have been proposed for the efficient destruction of miRNAs (Figures 2, 3). A comprehensive analysis of the efficiency of the developed anti-miRNA catalytic nucleic acids revealed that the ribonuclease activity of antimiRzymes and miRNases is influenced by several key factors: (1) the overall configuration of the anti-miRNA catalytic construct, including the structure of the binding domains, nature and composition of the catalytic component, the presence of metal ions necessary for catalysis, as well as the position of the catalytic domain towards a defined functional determinant of miRNA; (2) the sequence of the target miRNA; and (3) the patterns of modifications within the oligonucleotide structure. Supplementary Table S1 summarizes the structural features, ribonuclease characteristics and biological activity of the developed miRNA-targeted catalytic nucleic acids.

### 6.1 The overall configuration of catalytic oligonucleotides

For the development of antimiRzymes, researchers typically use classical models such as hammerhead ribozymes (Belter et al., 2016), 8–17 (Belter et al., 2016) and 10–23 DNAzymes (Jadhav et al., 2009; Kaur et al., 2010; Belter et al., 2016; Larcher et al., 2019; Wang et al., 2020), along with *de novo* selection of DNAzymes (Inomata et al., 2021; Donde et al., 2022) (Figure 2).

miRNases were constructed based on different oligonucleotide structures (linear, hairpin, dual, and bulge-forming) (Figures 3A–E), incorporating tris(2-aminobenzimidazole) (Danneberg et al., 2015), Lys-diethylenetriamine (Lys-DETA), short peptides such as HGG·Cu^2+^ (Gaglione et al., 2011) or cationic peptides with alternating leucine and arginine residues acetyl-(LRLRG)_2_ or (LR)_4_G-NH_2_ (Patutina et al., 2017a; Patutina et al., 2018; Patutina et al., 2019; Patutina O. et al., 2020; Patutina et al., 2022; Miroshnichenko et al., 2019a; Chiglintseva et al., 2024) as catalytic agents (Figure 3F).

Catalytic nucleic acids require optimal target-binding properties to ensure efficient cleavage while maintaining cycling catalysis, necessitating a delicate balance between specific binding and facile post-cleavage dissociation. In the design of antimiRzymes using an unmodified backbone, researchers typically employ binding arms comprising 7–15 nucleotides, with a total duplex length of 15–22 base pairs (Larcher et al., 2019; Wang et al., 2020; Inomata et al., 2021) (Figures 2A–C; Supplementary Table S1). The modifications of antimiRzymes (e.g., LNA) permit the use of shorter arms, ranging 6–9 nucleotides with a total duplex length of 12–15 base pairs (Jadhav et al., 2009; Kaur et al., 2010; Donde et al., 2022) (Figures 2D,E; Supplementary Table S1). Upon binding to a miRNA target, antimiRzymes typically form a 1–3 nucleotide gap, which serves as a potential cleavage site (Figure 2).

The construction of miRNases employs various oligonucleotide architectures, including linear, hairpin, dual, and bulge-forming structures (Figures 3A–E). Linear and hairpin miRNases typically incorporate a 10–16 nucleotide region complementary to the target miRNA, with the remaining single-stranded RNA regions available for catalytic attack (Gaglione et al., 2011; Danneberg et al., 2015; Patutina et al., 2017a) (Figures 3A,B,E; Supplementary Table S1). Hairpin miRNases contain a 12, 14 or 16-mer sequence complementary to miRNA and an elongated mini-hairpin GCGAAAGC (Figure 3B; Supplementary Table S1), recognized for its exceptional stability and resistance to nuclease degradation (Hirao et al., 1992; Yoshizawa et al., 1994). Such an addressing domain ensures the efficient binding and stabilization of the complementary complex with miRNA target (Patutina et al., 2017b). Dual miRNases contain two separate 8–10 nucleotide binding domains flanking the catalytic peptide, with strategic substitution of adenines by aminoadenines to enhance binding affinity (Patutina O. et al., 2020) (Figure 3C; Supplementary Table S1). Bulge-forming miRNases utilize a single 18-mer oligonucleotide that, upon binding, induces a 3-nucleotide loop in the miRNA sequence at the center of the duplex (Patutina et al., 2022) (Figure 3D; Supplementary Table S1).

Design of recognition domains is crucial in ensuring the targeted effect of the catalytic domain towards specific functional regions of miRNA, significantly influencing cleavage efficiency and miRNA inactivation in cellular and tissue environments.

The design of ribozymes and DNAzymes allows for targeted catalytic activity primarily on the central loop (Jadhav et al., 2009; Belter et al., 2016; Inomata et al., 2021; Donde et al., 2022), the 3′ supplementary interaction region (Jadhav et al., 2009; Kaur et al., 2010; Larcher et al., 2019; Wang et al., 2020; Inomata et al., 2021), and the 3′-terminal site of the seed region (7–8 nts) (Kaur et al., 2010; Larcher et al., 2019; Inomata et al., 2021) (Figure 2; Supplementary Table S1). miRNases can be designed to address all four functional determinants of miRNA (Figure 3; Supplementary Table S1). Linear and hairpin miRNases are engineered to bind to the core region and catalyze cleavage at the 3′-supplementary interaction and terminal regions (Gaglione et al., 2011; Danneberg et al., 2015; Patutina et al., 2017a) or seed and terminal regions as can be seen for crab-like miRNases, containing two peptides located at the 5′- and 3′-termini of miRNA (Chiglintseva et al., 2024). Catalytic peptides in dual and bulge-forming miRNases specifically target the central loop and 3′-supplementary interaction regions (Patutina O. et al., 2020; Patutina et al., 2022).

For the development of therapeutic catalytic nucleic acids, researchers focus on targeting well-established oncogenic miRNAs miR-372/miR-373 (Jadhav et al., 2009), miR-27a (Kaur et al., 2010), miR-21 (Belter et al., 2016; Patutina et al., 2017a; Patutina et al., 2018; Patutina O. et al., 2020; 2022; Larcher et al., 2019; Wang et al., 2020; Donde et al., 2022; Chiglintseva et al., 2024), miR-155 (Inomata et al., 2021), miR-17 (Patutina et al., 2018; Patutina O. et al., 2020; Patutina et al., 2022; Donde et al., 2022; Chiglintseva et al., 2024), miR-20a/b (Gaglione et al., 2011; Danneberg et al., 2015; Patutina et al., 2017a), miR-1323 (Gaglione et al., 2011) (Supplementary Table S1), which have been repeatedly shown to play crucial roles in the pathogenesis of various malignancies.

The study of ribonuclease activity of catalytic oligonucleotides is conducted under both single-turnover and multi-turnover reaction conditions. Single-turnover conditions assess the productivity of one catalytic cycle, while multi-turnover conditions evaluate the ability to cleave excess target molecules, simulating biological scenarios.

The efficiency of antimiRzymes largely depends on the nucleotide sequence of the catalytic domain (Figure 2; Supplementary Table S1). Classical model anti-miRNA 10–23 DNAzymes demonstrate high ribonuclease activity against miRNA, with the most active constructs achieving 50%–75% target cleavage within 1–2 h (Jadhav et al., 2009; Kaur et al., 2010; Belter et al., 2016) (Figures 2A,C; Supplementary Table S1). However, high efficiency typically requires large excesses of antimiRzymes and high concentrations of divalent metal ions (Mg^2+^, Ca^2+^, Zn^2+^ etc.) amounting to 10–25 mM for proper folding and catalysis – conditions incompatible with physiological environments. Reducing magnesium concentration results in a substantial loss of activity. When using 10 mM concentration of magnesium ions, a comparative analysis of the cleavage efficiency showed that 10–23 DNAzyme induces no more than 10%–15% cleavage of miR-21 in 1 h, while 8–17 DNAzyme demonstrates more than a threefold superiority in the rate of catalysis (Belter et al., 2016). Larcher et al. reported DNAzymes operating at 10 mM magnesium ion concentration, catalyzing 20%–40% cleavage of a 200-fold excess of miRNA within 2 h, clearly demonstrating the DNAzyme multi-turnover capability (Supplementary Table S1) (Larcher et al., 2019). Interestingly, while antimiRzymes proved to be effective in cleaving mature miRNAs, they showed reduced efficacy when targeting synthetic pre-miRNA (Jadhav et al., 2009; Kaur et al., 2010; Belter et al., 2016).

Despite these limitations, the promising results have spurred research into developing more advanced and biocompatible antimiRzymes. *De novo* selection of DNAzymes (Inomata et al., 2021; Donde et al., 2022) (Figures 2B,E; Supplementary Table S1) has yielded an improved design of catalytic core capable of effective miRNA degradation, reaching 70%–80% after 8 h at 1 mM concentrations of magnesium and zinc ions. The newly developed DNAzyme demonstrated a pronounced superiority in catalytic efficiency under conditions of reduced magnesium concentration (1 mM) compared to 8–17, 10–23 DNAzymes and hammerhead ribozyme_TLRB (Donde et al., 2022).

miRNases emerge as slower but much more stable and autonomous inhibitors under physiological conditions. DNA and PNA miRNases based on tris(2-aminobenzimidazole), used in excess with respect to the target, perform sequence-specific cleavage of miRNA, reaching 50% after 15 h (Figures 3A,F; Supplementary Table S1). Faster cleavage kinetics were demonstrated by HGG·Cu^2+^ and DETA-based miRNases. These ribonucleases performed efficient cleavage of miRNAs in an equimolar ratio. Cleavage reached a plateau value by 1 h and was 47.5% for the HGG conjugate and 90% for the DETA conjugate (Figures 3A,F; Supplementary Table S1). miRNase containing HGG peptide required Cu^2+^ as a cofactor to perform cleavage. The comprehensive analysis of ribonuclease activity across various structural variants of miRNases with acetyl-(LRLRG)_2_ peptide, developed by our research group, revealed that dual and bulge-forming conjugates (Figures 3C,D; Supplementary Table S1) exhibited the lowest cleavage efficiency (Patutina O. et al., 2020; Patutina et al., 2022), whereas linear, hairpin, and crab-like miRNases (Figures 3A,B,E) demonstrated superior characteristics, including those incorporating 2′-OMe modifications, achieving 70%–100% miRNA cleavage within 24 h (Patutina et al., 2017a; Patutina et al., 2019; Miroshnichenko et al., 2019a; Chiglintseva et al., 2024) (Supplementary Table S1). Notably, the most active structural variants demonstrated multi-turnover catalytic behavior: hairpin and crab-like conjugates achieved 75% and 100% degradation in 24 h, respectively, with a two-fold excess of target miRNA, indicating their robust capability for efficient substrate cleavage in a multi-turnover mode (Figures 3B,E; Supplementary Table S1). Hairpin ribonucleases have been shown to be able to efficiently cleave 5- and 10-fold excesses of miRNA, resulting in a total of 75% cleavage (Supplementary Table S1). Unlike DNAzymes, most miRNases demonstrate markedly reduced sensitivity to metal ions. Hairpin and dual miRNases were shown to exhibit magnesium-independent cleavage (Patutina et al., 2017a; Patutina O. et al., 2020), a characteristic that substantially broadens their therapeutic potential and practical applications.

A comparative analysis of cleavage efficiency for identical motifs across different functional regions of miRNA revealed that antimiRzymes targeting the central loop and 3′ supplementary interactions region (in particular AU or GU sites) exhibited superior performance, likely due to the formation of the most favorable duplex conformation in these regions (Kaur et al., 2010; Larcher et al., 2019).

The opposite picture is observed for miRNases: the terminal attachment of peptides at the 5′- and 3′-ends of the oligonucleotide domain, coupled with the formation of an extended heteroduplex spanning at least 13 nucleotides (as demonstrated in hairpin miRNase (Patutina et al., 2017a) and crab-like miRNase (Chiglintseva et al., 2024)), conferred a significant advantage over conjugates where the peptide was internally positioned within the oligonucleotide component, resulting in the formation of shorter duplex regions (illustrated by dual miRNases (Patutina O. et al., 2020) and bulge-forming miRNases (Patutina et al., 2022)).

The ribonuclease activity of miRNases can be substantially influenced by the number of catalytic peptides in the structure of miRNases. Investigation of the miRNases containing acetyl-(LRLRG)_2_ peptide as a catalytic domain showed that incorporation of two sequentially arranged peptides in the bulge-forming conjugates yields a four-fold enhancement in miRNA cleavage efficiency compared to single-peptide variants (Patutina et al., 2022) (Figure 3D; Supplementary Table S1). Even more striking results were observed with linear conjugates, where the introduction of two peptides with formation of a crab-like configuration produced a synergistic effect, resulting in a ten-fold acceleration of target degradation (Chiglintseva et al., 2024) (Figure 3E; Supplementary Table S1). Intriguingly, when peptides were introduced via abasic sugar residues in crab-like miRNases, the ribonuclease activity was significantly compromised, presumably due to constraints in peptide spatial mobility (Chiglintseva et al., 2024). While the presence of two catalytic peptides in the structure of miRNase markedly enhances its cleavage rates, addition of a third peptide not only fails to further improve ribonuclease activity, but results in lower efficiency compared to di-peptide variants, which was shown in studies utilizing model yeast tRNA (Staroseletz et al., 2020). These findings indicate that for bulge-forming and crab-like miRNases, a di-peptide architecture represents the optimal structural configuration.

### 6.2 The sequence of the target miRNA

The nucleotide sequence of a miRNA plays a key role in determining the optimal motifs for targeting by the catalytic domain of miRNA inhibitors and influences the rate of catalysis by XNAzymes and miRNases. The target cleavage sites must precisely align with nucleobase specificity of the catalyst and need to be located within the target region, specifically designed for the particular type of inhibitor.

For XNAzymes, where the 5′ and 3′ miRNA regions serve for binding, the presence of cleavage-sensitive sites within the central miRNA domain predominantly governs the catalyst efficiency. The design of 10–23 DNAzymes positions cleavage-sensitive purine-pyrimidine sites within the target gap region, enabling efficient catalysis by these nucleic acids (Jadhav et al., 2009; Kaur et al., 2010; Belter et al., 2016; Larcher et al., 2019; Wang et al., 2020) (Supplementary Table S1). Similarly, 8–17 DNAzymes target purine-purine sites, specifically A-G and G-G sequences, where their presence in the gap region is essential for successful RNA hydrolysis (Belter et al., 2016). Through *de novo* selection, Donde and colleagues engineered a DNAzyme capable of catalyzing miRNA cleavage specifically at U-U sites (Donde et al., 2022) (Supplementary Table S1). Inomata and co-authors developed a more versatile DNAzyme that is able to catalyze cleavage at every linkage in a dinucleotide targeted gap – U-A, A-A, and U-G (Inomata et al., 2021) (Supplementary Table S1). These findings highlight the structural flexibility of DNAzymes, which can be precisely engineered to achieve diverse nucleotide specificities through careful consideration of target miRNA sequences.

Depending on the nature of the employed catalytic domain, synthesis conditions, and overall structural configuration, miRNases demonstrate distinct nucleotide cleavage specificity, thus their catalytic activity is critically dependent on the target miRNA sequence. The influence of miRNA sequence is distinctly observed for miRNases containing acetyl-(LRLRG)_2_ or (LR)_4_G-NH_2_ peptides as scissor components. In particular, studies of bulge-forming miRNases with Pyr-X-specific cleavage showed that a shift in the trinucleotide loop, formed upon binding, leads to the exposure of sensitive C-A bonds and results in a 1.5-fold enhancement of miRNA cleavage (Patutina et al., 2022). Dual miRNases demonstrated similar sequence sensitivity: the introduction of cleavage-sensitive sites into the gap region increased cleavage efficiency by up to 20% (Patutina O. et al., 2020). The sequence effect was also evident in hairpin and linear miRNases, including crab-like conjugates. Structurally identical ribonucleases targeting miR-21 and miR-17 exhibited distinct cleavage kinetics (Patutina et al., 2018; Chiglintseva et al., 2024) (Supplementary Table S1, comparison of crab-α-21 and crab-α-17). This difference arises from the distribution of cleavage-sensitive C-A and U-A bonds, which are present in the 5′- and 3′-single-stranded regions of miR-17 but absent in the terminal regions of miR-21.

PNA-based miRNases incorporating tris(2-aminobenzimidazole) (Danneberg et al., 2015), Lys-DETA, and short peptide HGG·Cu^2+^ (Gaglione et al., 2011) demonstrate Pur-Pur specificity in RNA cleavage. Danneberg *et al*. showed that pyrimidine bases at the cleavage site significantly reduce substrate cleavage efficiency. Additionally, due to PNA conjugates tendency to form aggregates, the authors emphasize the necessity of optimizing PNA oligomer length, especially for G/C-rich chains. This further highlights the importance of careful miRNA sequence analysis for construction of efficient nucleases.

### 6.3 Modification pattern of the oligonucleotide

Chemical modifications in the structure of catalytic nucleic acids can profoundly affect their biological properties. Strategic introduction of chemical modifications into the structure of catalytic nucleic acids offers a pathway to enhance their enzymatic cleavage properties through increased affinity for miRNA targets and stabilization of their active structural configuration, as well as to improve their biological efficiency by increasing resistance to nucleases in biological environments.

For antimiRzymes, it was revealed, that incorporating of 2–4 LNA units into the miRNA-binding regions (arms) of 10–23 DNAzymes led to an approximately twofold increase in the rate of miRNA cleavage (Figure 2D; Supplementary Table S1). This highlights the enhanced affinity and potency of LNA-modified antimiRzymes compared to their unmodified counterparts (Jadhav et al., 2009; Kaur et al., 2010). Donde *et al*. engineered DNAzymes with fully 2′-deoxy-2′-fluoro-β-D-arabino nucleic acid (FANA) backbones (Figure 2E; Supplementary Table S1), enabling the enzymes to retain catalytically active configurations even at reduced Mg^2+^ concentrations and providing additional stability in biological conditions (Donde et al., 2022).

Peptide nucleic acid (PNA) has garnered the attention of some research groups as a potential binding domain in artificial ribonucleases (miRNases), owing to its unique hybridization properties and notable chemical and biological stability (Gaglione et al., 2011; Danneberg et al., 2015) (Figure 3A). To overcome certain obstacles associated with the formation of hydrophobic aggregates that limit water solubility, PNA oligomers can be conjugated with cationic amino acids, cell-penetrating peptides, or hydrophilic polymers. Gaglione *et al*. demonstrated improved solubility and effective target miRNA cleavage by incorporating polyethylene glycol (PEG) units and lysine residues into miRNases with HGG·Cu^2+^ and Lys-DETA cleaving groups. Notably, conjugates without PEG showed significantly reduced processivity (Gaglione et al., 2011). Comparative analysis of tris(2-aminobenzimidazole)-based miRNases with DNA or PNA oligonucleotide domains revealed similar ribonuclease activity against target miRNA (Danneberg et al., 2015). However, higher affinity of PNA analogue to RNA substrates enabled the use of shorter oligomers (10- and 15-mers). PNA variants of miRNases are hypothesized to exhibit enhanced efficacy under physiological conditions due to their increased nuclease resistance, suggesting promising potential for therapeutic applications.

Studies of dual miRNases containing the acetyl-(LRLRG)_2_ peptide demonstrated that substitution of adenines with amino-adenines in the 8–10 nucleotide oligonucleotide arms significantly enhances the affinity of the nuclease towards its target miRNA and consequently the cleavage efficiency (Patutina O. et al., 2020). Our studies on hairpin miRNases have also revealed intriguing insights into the effects of oligonucleotide modifications on miRNA cleavage efficiency. Complete 2′-O-methylation (2′-OMe) of the oligonucleotide component was found to reduce miRNA cleavage efficiency. However, a strategic partial substitution of the DNA backbone with 2′-OMe residues, leaving three nucleotides unmodified near the peptide attachment site, significantly enhances the cleavage rate (Miroshnichenko et al., 2019a) (Figure 3B; Supplementary Table S1).

## 7 Synergy in action: collaborating with RNase H for enhanced efficacy

**FIGURE 4:**
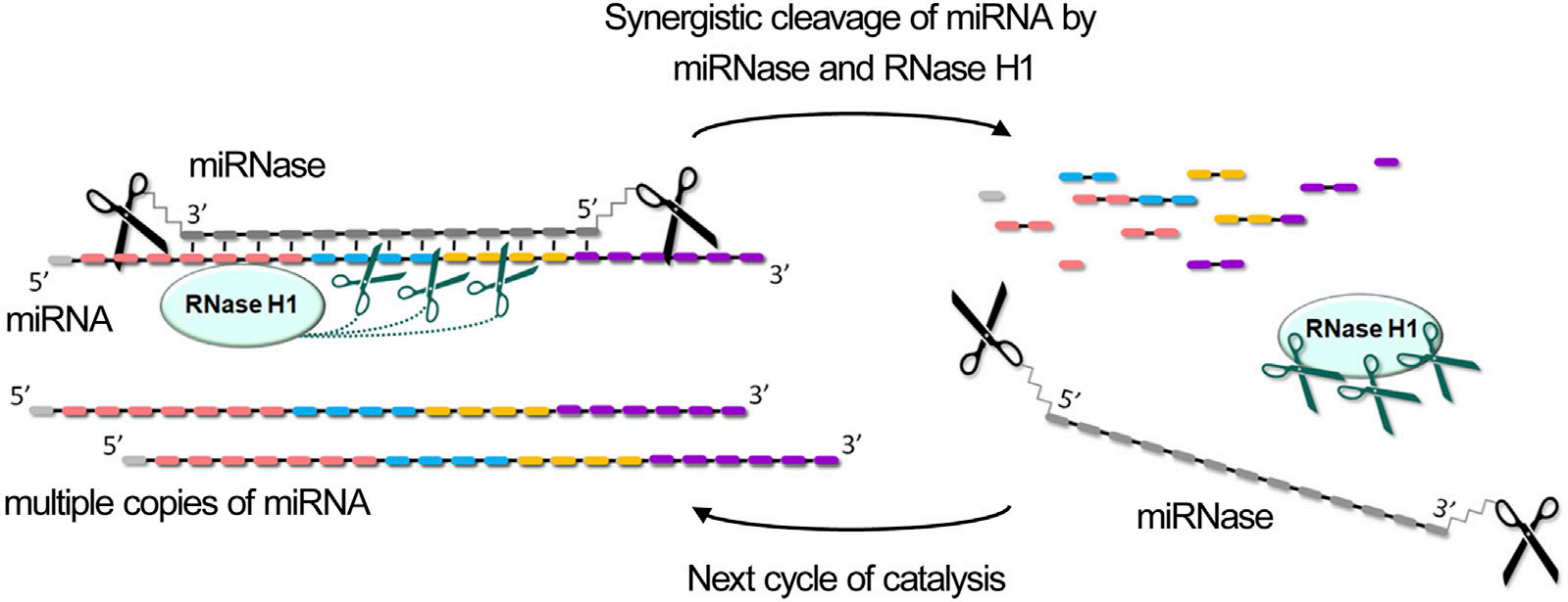
Synergistic degradation of miRNA through coordinated enzymatic cleavage by miRNase and RNase H. The combined action of miRNase and RNase H1 results in the degradation of miRNA across all functional domains, generating 2-3-nucleotide fragments, which leads to their rapid dissociation and releasing of miRNase and RNase H1 for the next catalytic cycle.

One of the fundamental mechanisms underlying antisense-based RNA suppression in cellular systems is the selective degradation of target RNA molecules in DNA heteroduplexes through RNase H-mediated cleavage. In addition to the inherent ribonuclease activity, catalytic RNA inhibitors containing natural unmodified DNA oligonucleotides as RNA-recognition domains can potentially recruit cellular RNase H to cleave RNA within DNA hybrids, substantially enhancing miRNA degradation efficiency. Indeed, one of the exceptional characteristics we have established for miRNases, bearing acetyl-(LRLRG)_2_ or (LR)_4_G-NH_2_, is their pronounced synergy with RNase H. By mimicking intracellular conditions, it was shown that heteroduplexes of miRNase and miRNA act as substrates for RNase H, enabling additional RNA cleavage within the paired region (Figure 4).

The combined action of miRNase and RNase H provides record acceleration of cleavage rate, achieving 10–30 fold increases compared to either enzyme alone, depending on the type of miRNase (dual, bulge-forming, hairpin, or crab-like), excess of miRNA in the reaction, the concentration of RNase H, and target sequence composition (Patutina et al., 2018; Patutina O. et al., 2020; Patutina et al., 2022; Chiglintseva et al., 2024). What is especially remarkable, RNase H proves particularly valuable when employing miRNases that do not exhibit multiple turnover catalysis *in vitro*, such as dual and bulge-forming types. For such miRNases, the synergistic effect is even higher than for more active variants, bringing them closer in terms of efficiency when intended for application *in vivo*. This dual-enzyme cleavage mechanism provides a distinct advantage by degrading miRNA throughout its entire length, targeting all functionally significant regions: single-stranded areas are cleaved by miRNases while the heteroduplex regions are degraded by RNase H (Patutina et al., 2018; Patutina O. et al., 2020; Patutina et al., 2022; Chiglintseva et al., 2024) (Figure 4).

The synergistic enhancement of RNA degradation through combined action of the conjugate and RNase H can be attributed to: (a) prevention of peptide non-productive conformations through RNase H interaction with the heteroduplex, enhancing conjugate activity; (b) better heteroduplex stabilization by the conjugate compared to oligonucleotide alone, leading to more efficient RNase H substrate cleavage; (c) increased processivity due to easier dissociation of both the conjugate and RNase H from fragmented RNA targets for subsequent cleavage cycles. Additionally, recent studies have shown that certain proteins can bind to and enhance RNase H activity (Zhang et al., 2022). It can be hypothesized that the peptide component of miRNases may similarly possess properties that potentiate RNase H activity, further contributing to the observed synergistic effects.

DNAzymes with binding arms exceeding 7-8 nucleotides have a structural organization where the duplex regions formed with target miRNAs can potentially serve as RNase H substrates. Similar to miRNases, this dual functionality may lead to synergistic enhancement of RNA cleavage rates. However, this RNase H-mediated enhancement is not universal across all designs: XNAzymes with shorter binding arms and intramolecular modifications that do not confer RNase H substrate properties cannot utilize this mechanism. Studies using synthetic model RNA substrates have demonstrated that the concurrent action of RNase H and DNAzymes with 10–12 nucleotide binding arms substantially accelerates target degradation (Dubovichenko et al., 2024; Kolpashchikov and Gerasimova, 2025). While the combined effects of miRNA-targeted DNAzymes and RNase H have not yet been experimentally investigated, there is a strong theoretical basis to anticipate enhanced target miRNA degradation efficiency.

The synergy with RNase H demonstrates global significance by expanding the potential for applying catalytic nucleic acids within living systems, ensuring effective inactivation of target miRNAs and potentially blocking pathological processes in cancer cells. From a therapeutic perspective, the most promising catalytic nucleic acids are those whose RNA recognition elements incorporate chemical modifications that provide crucial benefits such as enhanced hybridization properties, resistance to nuclease degradation, and preserved RNase H functionality. For instance, in dual miRNases, the substitution of natural adenines with 2′-aminoadenines in the RNA-binding oligonucleotide sequence both strengthens target miRNA binding and maintains RNase H recruitment capabilities (Patutina O. et al., 2020). Emerging chemical modifications show particular promise, including 5′-O-Methylphosphonate (Šípová et al., 2014), mesyl-N-(methanesulfonyl)-phosphoramide modifications (Miroshnichenko et al., 2019b; Patutina O. A. et al., 2020), and strategic gapmer architectural designs (Egli and Manoharan, 2023).

## 8 Unlocking the power: biological activity of catalytic nucleic acids

Recent advances have produced catalytic agents for sequence-specific miRNA degradation, serving as valuable RNA manipulation tools in laboratory research. Beyond experimental applications, such engineered enzymatic molecules show particular therapeutic promise through their selective multi-turnover mechanism against pathogenic RNA molecules. This repetitive catalytic mechanism enables the use of minimal doses of these oligonucleotide compounds while effectively eliminating multiple copies of disease-associated targets.

Several successful examples of miRNA suppression in eukaryotic cells using antimiRzymes have been reported. The assessment of the biological activity of antimiRzymes and miRNases in tumor cells has been predominantly investigated using the widely used commercial transfection agent Lipofectamine, although some studies have explored aptamer-mediated delivery systems, which offer the potential for more targeted and specific cellular uptake (delivery methods are listed in the Supplementary Table S1). Maiti’s group evaluated the biological effects of 10–23 XNAzymes in HEK293 cells that were consistently transfected with a vector expressing miR-372/miR-373 (Jadhav et al., 2009) or miR-27a (Kaur et al., 2010), followed by XNAzyme delivery. The obtained results demonstrated that DNAzymes achieved a 35%–40% reduction in miRNA target levels, while LNAzymes (DNAzymes containing 2 or 4 LNA units) showed higher efficacy with 50%–60% suppression. When cells were additionally transfected with luciferase vector containing 3′-UTR binding sites of direct target genes of the studied miRNAs – LATS2 (miR-372/miR-373) (Jadhav et al., 2009) and prohibitin (miR-27a) (Kaur et al., 2010) – miRNA expression was found to suppress target product synthesis, whereas XNAzyme treatment led to a 1.5-2-fold increase in target product levels (Supplementary Table S1). Notably, XNAzymes containing mutations in their catalytic sequence showed no effect on miRNA levels or their target genes. In studies (Belter et al., 2016; Larcher et al., 2019), the authors investigated the biological potential of hammerhead ribozymes and 10–23 DNazymes for suppressing endogenous miR-21 in glioblastoma cells T98G, U87MG and breast carcinoma cells MDA-MB-231. Transfection of ribozyme and DNAzyme into T98G cells resulted in 80% and 50% reduction in miR-21 levels, respectively, and increased levels of its direct target protein PTEN by 4.3- and 3.1-fold, respectively (Belter et al., 2016) (Supplementary Table S1). The suppression of miR-21 in U87MG glioblastoma and MDA-MB-231 cells by the most active 10–23 DNAzyme showed dose-dependent silencing. Maximum miR-21 reduction reached 90% in U87MG cells (200 nM) and 54% in MDA-MB-231 cells (400 nM) (Larcher et al., 2019). The authors also developed a DNAzyme conjugate with a transferrin receptor targeting aptamer, which provided efficient delivery and achieved 52% suppression of miR-21 in U87MG cells (2 µM) (Supplementary Table S1). Similarly, Wang *et al*. demonstrated effective miR-21 reduction using a 10–23 DNAzyme conjugated with an LDL-R-recognizing aptamer, achieving 56% suppression in Huh-7 liver cancer cells (2 µM) (Wang et al., 2020) (Supplementary Table S1). It is worth noting that not all studies involving DNAzymes and their derivatives adequately describe and implement necessary controls. Consequently, the observed reduction in intracellular RNA levels might be attributed to antisense effects – either through steric blocking or RNase H-mediated degradation – rather than the actual catalytic activity of DNAzymes.

Among the developed miRNases, only metal independent peptide-based compounds containing acetyl-(LRLRG)_2_ and (LR)_4_G-NH_2_ have been extensively studied for their biological potential in tumor cells, demonstrating significant miRNA-inhibiting activity across different tumor cell lines. The hairpin miRNase containing acetyl-(LRLRG)_2_ peptide achieved a 50% reduction in miR-21 levels in RLS_40_ lymphosarcoma cells, resulting in a 1.7-fold and 2.4-fold increase in the level of direct targets PTEN and PDCD4, respectively, while inhibiting proliferative activity (Patutina et al., 2017a; Patutina et al., 2019) (Supplementary Table S1). In the B16 melanoma cell model, this miRNase induced apoptosis in 28% cells (vs. 4.5% in the control) and inhibited invasion capacity of tumor cells by 70% (Patutina et al., 2019) (Supplementary Table S1). The hairpin miRNase containing (LR)_4_G-NH_2_ peptide demonstrated dose-dependent inhibition of miR-17 in B16 melanoma cells, achieving 40% and 80% reduction at 100 nM and 500 nM concentrations, respectively (Patutina et al., 2018) (Supplementary Table S1), leading to a 2.2-fold increase in the level of its direct target E2F1 and 5-fold inhibition of cell proliferation. In MCF7 breast carcinoma cells, crab-like miRNases effectively inhibited miR-21 and miR-17 by 65% and 58%, respectively, resulting in a 1.7-fold increase in PDCD4 and a 1.9-fold elevation of E2F1 expression, ultimately leading to 50% inhibition of proliferation (Chiglintseva et al., 2024) (Supplementary Table S1). A favorable characteristic of biologically active miRNases that ensures their highly effective action in cells is their unique structure, which provides intramolecular nuclease protection through flanking of the oligonucleotide domain either by two peptides in crab-like conjugates, or by a hairpin structure at the 3′-end and a peptide at the 5′-end in hairpin miRNases, extending nuclease stability up to 8 and 48 h, respectively (Patutina et al., 2018; Chiglintseva et al., 2024). To demonstrate the importance of the catalytic nature of miRNases action, comparison with corresponding peptide-free oligonucleotides clearly demonstrates that the catalytic activity of the miRNase peptide component plays a crucial role in ensuring efficient miRNA inactivation (Patutina et al., 2017a; 2018; Patutina et al., 2019; Patutina O. et al., 2020; Patutina et al., 2022; Chiglintseva et al., 2024).

The performance metrics of developed miRNA-targeted catalysts are comparable to or superior to existing miRNA inhibition technologies. For instance, they surpass the efficacy of traditional 2′-OMe antisense oligonucleotides, which achieve а 50% reduction in glioblastoma invasion (Dong et al., 2012) and provide similar anti-migrative effects as PMO-anti-miRNA oligonucleotides that exhibit about 45%–60% downregulation of colorectal cancer cells motility (Yu et al., 2023). Moreover, miRNA-targeted cleavers demonstrate comparable or superior *in vitro* performance in contrast to a recently engineered chimeric construct, consisting of miRNA-targeted antisense oligonucleotide, RNase L-recruiting moiety and an aptamer for targeted delivery. Designed compounds provide RNase L-mediate cleavage of miRNA targets in the cells resulting in 33%–50% reduction in breast cancer cell viability (Fang et al., 2024). When compared to commercial solutions, engineered artificial ribonucleases demonstrate superior outcomes, exceeding the performance of both the Ambion inhibitor, which achieves 50% invasion reduction and 15% apoptosis induction in esophageal cancer cells (Wu et al., 2016), and the GenePharma product, which results in 50% proliferation inhibition in pancreatic cancer cells (Li et al., 2017).

Based on the obtained data, analysis of structural assemblies reveals that among antimiRzymes, the variants demonstrating highest efficiency are those with catalytic activity targeted to the central loop region and 3′ supplementary interaction sites of miRNA sequences. In contrast, among miRNases, superior therapeutic characteristics are exhibited by conjugates whose nuclease activity targets the terminal regions of miRNAs, specifically the seed and terminal regions including the complementary interaction sites. This effect may be attributed to more efficient recruitment of RNase H when forming an extended unbroken duplex involving the central region of miRNA.

For *in vivo* applications, the universal challenges facing nucleic acid-based therapeutics, including nuclease degradation susceptibility, inefficient intracellular delivery and trafficking, can be addressed through the introduction of an optimized set of modifications to the oligonucleotide sequence of engineered enzymes, while preserving their catalytic activity. However, a major additional constraint in the therapeutic application of antimiRzymes lies on their stringent requirement for high concentrations of divalent metal ions to achieve proper folding, catalysis, and cleavage in biological systems. The intracellular environment cannot provide metal ion concentrations necessary for optimal XNAzyme performance, consequently, observed effects may predominantly arise from antisense activity rather than catalytic function. Nevertheless, recent advances in nanomedicine have enabled the development of nanocarriers incorporating metallic ion cofactors, whose rational design may significantly enhance both efficient delivery and activity of DNAzyme *in vivo* (Liu et al., 2021; Yan et al., 2023a). Despite certain evident limitations of the technologies discussed, the current results demonstrate considerable promise for the developing miRNA-targeted catalytic inhibitors. Notably, the majority of miRNases do not require metal ions for their activity, making their therapeutic potential a promising area of research.

## 9 The antitumor potential of catalytic nucleic acids

To advance the development of catalytic miRNA-inhibitors further, investigating their antitumor activity in living organisms represents a particularly crucial task. Acetyl-(LRLRG)_2_ peptide-based miRNases represent pioneering catalytic nucleic acids with demonstrated antitumor effects. For evaluation of antitumor activity in mouse models, miRNases were delivered into tumor cells via Lipofectamine transfection under *in vitro* conditions, followed by *in vivo* tumor growth assessment. Tumor growth analysis revealed that a single treatment of tumor cells *in vitro* with hairpin miRNase resulted in 95% suppression of RLS_40_ lymphosarcoma growth in mice, demonstrating superiority over the antisense effects of different types of corresponding peptide-free oligonucleotides (Patutina et al., 2019) (Supplementary Table S1). The most active conjugate in the series of crab-like conjugates also exhibited high potency, achieving 85% reduction in MCF7 tumor growth compared to the non-specific control conjugate. The less active crab-like variant also demonstrated substantial antitumor activity but with reduced antitumor effect (50%) (Chiglintseva et al., 2024) (Supplementary Table S1). These results strongly support the mechanistic importance of ribonuclease activity, as higher catalytic activity correlates directly with enhanced antitumor effects. Histological and immunohistochemical analyses of tumor tissues revealed that the antitumor effect was characterized by a three-fold reduction in both mitotic and proliferative activities of MCF7 tumors (Chiglintseva et al., 2024).

It should be noted that in terms of antitumor effect, miRNases are comparable to or outperform various modified oligonucleotides containing phosphorothioate (PS), 2′-O-methoxyethyl (2′-MOE), 2′-fluoro (2′-F), or constrained Ethyl nucleic acids (cEt) modifications, which show 40%–80% tumor growth inhibition in hepatocellular carcinoma models *ex vivo* (Wagenaar et al., 2015).

Collectively, these findings demonstrate the remarkable therapeutic potential of catalytic miRNA inhibitors, particularly peptide-based miRNases, and lay a strong foundation for further development of catalytic miRNA inhibitors as innovative antitumor therapeutics. Although anticancer applications of DNAzymes remain relatively unexplored, these molecules represent a highly advanced technology that is rapidly evolving towards the generation of multifunctional nanoscale machines for the comprehensive suppression of pathological molecules, thus allowing us to anticipate highly efficient catalytic constructs for the suppression of miRNAs in the near future.

## 10 Mechanisms and advantages of miRNA-targeted catalytic nucleic acids

**FIGURE 5:**
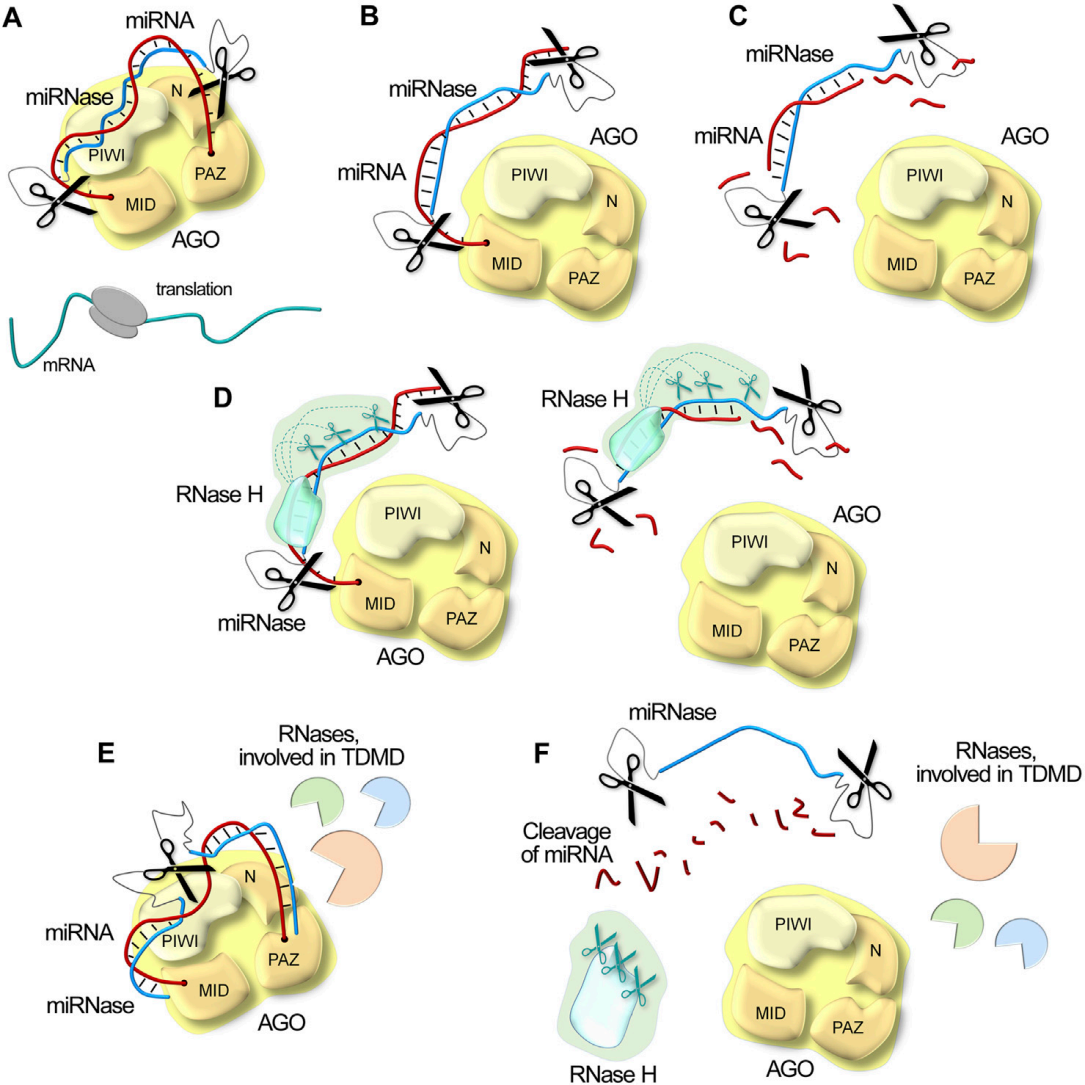
Schematic illustration of possible mechanisms of miRNA inactivation in the cell under the action of miRNases/antimiRzymes, RNase H and intracellular RNases involved in Target-Directed MicroRNA Degradation (TDMD). **(A)**. Steric blocking of miRNA through complementary binding. Upon miRNA blockage, the mRNA escapes repression, thereby enabling efficient protein translation. **(B)**. Displacement of miRNA from the AGO complex. **(C)**. Site-specific degradation of miRNA by catalytic domains of miRNases/antimiRzymes. **(D)**. Synergistic degradation of miRNA by combined action of miRNases/antimiRzymes and RNase H, resulting in acceleration of RNA degradation kinetics. **(E)**. Cellular degradation pathway activation: miRNases/antimiRzymes can engage cellular machinery driving TDMD with additional recruitment of other intracellular nucleases. **(F)**. Degradation of miRNA target in all functional determinants by multi-modal mechanism, combining both direct catalytic activity and cellular pathway engagement.

The remarkable capabilities of catalytic nucleic acids lie in their sophisticated multi-modal approach to miRNA inhibition. In contrast to canonical antisense oligonucleotides, XNAzymes and artificial ribonucleases represent autonomous molecular agents, engaging several parallel mechanisms and creating a powerful strategy for RNA regulation. The proposed mechanisms of action of miRNA-targeting catalytic nucleic acids and their advantages will be outlined below (Figure 5).

At the most fundamental level, catalytic nucleic acids employ (1) direct physical interference. By analogy with antisense oligonucleotides, these inhibitors complementarily bind miRNAs (Figure 5A), creating a steric blocking effect that prevents miRNA function. Complementary binding not only blocks the miRNA but also forces its (2) displacement from the RISC (Figure 5B). Further, the catalytic moiety of inhibitors provides (3) direct cleavage of miRNA targets (Figure 5C). A particularly noteworthy advantage of artificial RNases emerges from their independence from endogenous RNase H activity. This autonomous functionality sets catalytic nucleic acids apart from traditional antisense oligonucleotides and opens up new possibilities for molecular design. Scientists can freely introduce chemical modifications to the oligonucleotide domain, addressing crucial aspects such as stability, cellular delivery, and transportation without compromising functionality. This independence becomes especially valuable in biological fluids lacking RNase H, such as in cancer scenarios where elevated circulating oncogenic miRNAs drive disease progression.

The story becomes even more interesting when we consider oligonucleotides with RNase H-recruiting properties as binding domains. In these cases, miRNases (4) work in cooperation with RNase H (Figure 5D), creating a synergistic effect that dramatically accelerates RNA degradation kinetics. This conjoint action represents a significant advancement in RNA-targeting strategies. Finally, catalytic oligonucleotides, in particular such structures, as DNAzymes and dual or bulge-forming miRNases, can (5) engage cellular machinery that drives Target-Directed MicroRNA Degradation (TDMD) – the main mechanism of intrinsic miRNA degradation. When hybridizing with specific targets, catalytic oligonucleotides can effectively mimic natural target interactions involved in TDMD, potentially triggering the inner miRNA degradation pathways and recruiting various intracellular nucleases to assist the cleavage process (Figure 5E).

Due to their multimodal action, miRNA-targeting catalytic nucleic acids can provide efficient degradation of miRNAs (Figure 5F) and offer advantages over physical blocking or sequestering oligonucleotides. A single catalytic molecule can undergo multiple turnover cycles, cleaving numerous copies of target RNA, thereby enhancing both reaction kinetics and duration of action while reducing required dosages and minimizing toxic effects.

The advantages of catalytic miRNA degradation compared to steric blocking mechanisms may be particularly valuable in therapeutic contexts. Catalytic nucleic acids function as highly specific inhibitors, and rational incorporation of chemical modifications enables the design of compounds with exceptional target specificity. This precision is usually achieved by introducing modifications within binding domains that prevent recruitment of RNase H1, ensuring exceptionally site-specific cleavage while avoiding non-specific degradation at the binding region (Liu et al., 2025), which is a critical requirement for inactivating mutant miRNA forms or selectively targeting specific miRNAs within multimember families. Furthermore, degradation-based miRNA inhibitors exhibit reduced cellular toxicity since target miRNA degradation facilitates RISC complex recycling and preserves its functionality. Conversely, when therapeutic objectives involve eliminating aberrant RNA molecules and destroying pathogenic cells, recruitment of RNase H1 activity can promote extensive target cleavage, resulting in irreversible loss of functional activity, enhanced catalytic turnover through dual functionality, improved therapeutic efficacy, and complete destruction of pathological foci. Collectively, these advantages establish catalytic nucleic acids as promising therapeutic tools.

## 11 Challenges and future perspectives

The emerging technologies of miRNA catalytic inactivation, including antimiRzymes and miRNases, represent promising therapeutic approaches. These strategies offer the undeniable advantage of enabling lower dosage treatments, which could substantially reduce toxic burdens while maintaining therapeutic efficacy. In the field of developing approaches for the catalytic destruction of miRNAs, efforts are also focusing on the development of bioengineered molecular instruments based on RNA-binding proteins (Ahmed et al., 2023) for substrate-specific miRNA destruction, which further highlights the potential of this research field.

Despite their high therapeutic potential, preclinical and clinical application of miRNA-targeted catalytic nucleic acids remains limited due to several unresolved challenges, including stability, delivery, circulation time and pharmacokinetics, off-target and potential adverse effects, and dependence on metal ion concentrations.

Effective delivery of nucleic acids to target cells and tissues still remains an unresolved challenge. The delivery of DNAzymes, ribozymes, and artificial ribonucleases employs established approaches developed for antisense oligonucleotides, such as cationic lipids, copolymer-based nanoparticles, and inorganic nanomaterials (Xiao et al., 2023). However, these carriers are frequently associated with significant off-target toxicity, inefficient compound encapsulation, and slow endosomal escape. These limitations may be addressed by employing dendrimers, metal-organic frameworks (MOFs), and DNA nanomaterials. Specifically, MOFs enable precise coordination and delivery of metals to tumor sites *in vivo*, while DNA-based nanostructures, such as tetrahedral and capsule-like assemblies, allow programmed release of DNAzymes into cells, thereby improving pharmacokinetics (Wang et al., 2019; Wang J. et al., 2021). Nevertheless, the clinical translation of such constructs may be hindered by their large size. An alternative approach combining efficient delivery and nuclease resistance involves chitosan-based hydrogels, which have demonstrated high efficacy in intradermal DNAzyme administration (Eicher et al., 2019). Another strategy is the conjugation of catalytic nucleic acids with aptamers or short peptide fragments (e.g., cell-penetrating peptides, CPPs), enabling targeted delivery without additional carriers (Li et al., 2020). However, this design must account for potential reductions in catalytic activity and target affinity due to misfolding of DNAzyme tertiary structures (Liu et al., 2023). Similarly, artificial ribonucleases can be delivered without auxiliary agents by incorporating additional positively charged amino acids into their catalytic peptide domains. Yet, this approach requires careful optimization to prevent aggregation caused by electrostatic inter- and intramolecular interactions and reduction of catalytic activity.

Enhanced pharmacokinetic properties of catalytic nucleic acids can be achieved through various factors, including improved nuclease resistance and extended circulation time, which can be accomplished by introducing specific chemical modifications. Phosphorothioate (PS) linkages enhance biological stability, cellular uptake, and pharmacokinetic properties, though they may cause nonspecific protein binding, immune responses, and reduced catalytic activity (Zhao et al., 1996; Schubert et al., 2003; Chakravarthy et al., 2017). Additional modifications such as FANA, LNA, and 2′-OMe in recognition arms improve RNA affinity and nuclease resistance, although excessive affinity may impede substrate release and reduce catalytic turnover (Larcher et al., 2023). Modifications recruiting RNase H1, such as PS, mesylphosphoramide, 5′-O-methylphosphonate for irreversible RNA degradation, may significantly increase not only nuclease resistance, but also reaction turnover and consequently consider to be more advantageous. However, modifications within the catalytic core of DNAzymes or near catalytic group attachment sites in miRNases require careful consideration, as minimal structural changes can disrupt active conformation and dramatically reduce catalytic activity (Miroshnichenko et al., 2019a; Wang Y. et al., 2021).

The potential toxicity of catalytic nucleic acids represents another important issue and can be associated with several factors including sequence composition, chemical modifications, dosage, and delivery route. DNAzymes and ribozymes exhibit catalytic activity only upon binding to their specific substrates through precise complementary base pairing (Santoro and Joyce, 1998; Peng et al., 2021; Xiao et al., 2023). Therefore, off-target effects associated with the catalytic component are expected to be minimal. However, the cofactor requirement for high concentrations of metal ions may disrupt cellular ionic homeostasis, potentially leading to undesirable adverse effects. The catalytic domain of peptide-based miRNases represents a short synthetic peptide chain that intrinsically exhibits low immunogenicity due to the absence of molecular patterns required for recognition by dendritic cells and macrophages (Alharbi et al., 2022). However, potential off-target effects of catalytic components of miRNases require further investigation.

Beyond catalytic activity, the nucleic acid foundation of these molecules introduces additional safety considerations. Since these therapeutic agents are nucleic acid-based, they may exhibit nonspecific effects including hybridization-dependent off-target interactions and sequence-dependent immunogenic responses (Goyenvalle et al., 2023). While computational approaches can address off-target effects for longer mRNA targets through predictive analysis of oligonucleotide interactions (Hagedorn et al., 2017), this strategy remains challenging for short miRNA targets, necessitating thorough target selection with strictly therapeutic implications and development of highly targeted delivery systems.

The application of chemical modifications can further complicate the safety landscape. Along with enhancement of biological stability, circulation time, and pharmacokinetic properties, they may simultaneously induce unwanted immune reactions and adverse effects (Goyenvalle et al., 2023). Chemical modifications have been strategically employed to modulate undesirable immunogenicity, specifically, phosphorothioate modifications are known to enhance immune stimulation (Liang et al., 1996; Zhao et al., 1996), whereas 2′-O-methyl modifications and ribose alterations, such as 2′-F, 2′-MOE and LNA, effectively reduce inflammatory responses (Henry et al., 2000; Sewing et al., 2017). Nevertheless, studies have demonstrated that phosphorothioate and LNA modifications can lead to serious toxic effects including hepato- and nephrotoxicity, thrombocytopenia, and coagulopathy (Goyenvalle et al., 2023). To address these issues, strategic incorporation of single selective substitutions into the sequence of catalytic nucleic acids may produce an optimal product.

Current clinical trials with DNAzymes provide valuable insights into their safety profile, though evaluation remains limited to mRNA target suppression for chronic inflammatory and oncological diseases. These studies have predominantly included DNAzymes containing 3′-3′-inverted dT modifications, which provide protection against exonucleases and extend serum half-life (Dass et al., 2002; Li Y. et al., 2022; Yan et al., 2023b). Clinical data demonstrate excellent safety profiles of DNAzymes without inducing adverse effects or immune responses, establishing them as promising therapeutic candidates (Yan et al., 2023b). However, despite good safety profiles, clinical trials indicate modest *in vivo* efficacy, attributed primarily to suboptimal conditions, particularly the requirement for high cofactor concentrations. Current research focuses on resolving this limitation through two design strategies: developing enzyme modifications that function effectively at lower metal ion concentrations and creating self-regulating nanomachines capable of autonomously managing metal ion levels within catalytic centers, such as DNA nanocapsules or MOFs (Wang et al., 2019; Wang J. et al., 2021). In contrast, miRNases show greater compatibility with physiological environments, which positions them as promising candidates for preclinical and clinical investigations.

An additional key barrier impeding the widespread clinical implementation of catalytic nucleic acid-based therapeutics is large-scale synthesis due to limited manufacturing capacity and high production costs. This is particularly problematic for molecules with mixed backbones containing complex chemical modification patterns. Additionally, nucleotide errors during synthesis pose a critical concern, as they can severely impair catalytic activity when occurring within the catalytic core region, while also affecting specificity and overall efficacy. The synthesis of artificial ribonucleases presents further complications for large-scale production due to their more complex hybrid structure, requiring additional conjugation steps with catalytic moieties. The development of optimal synthesis protocols and prevention of aggregation still remain ongoing challenges. Furthermore, purification procedures require additional optimization to increase product yield and eliminate undesirable reaction components, which may adversely affect the formation of catalytically active conformations of the final drug products. Concurrently, the synthesis of delivery carriers presents additional hurdles for large-scale implementation, as promising delivery systems demonstrating high *in vitro* efficacy appear to be too complex and costly for industrial production.

Despite unresolved challenges, catalytic nucleic acids may serve as the foundation for effective therapeutic agents. The strategic use of minimal selective modifications combined with the crucial advantage of low dosing offers a promising path toward developing safe and effective miRNA-targeted catalytic therapeutics. With these advantages and the rapid development of advanced nanotechnology delivery platforms, catalytic nucleic acids represent a highly promising area in nucleic acid medicine with strong potential for future clinical use.
