# Supplementary material for: Opening new frontiers with catalytic nucleic acids in miRNA inhibition
Source: Front Pharmacol. 2025 Jun 23;16:1604711. doi: 10.3389/fphar.2025.1604711 (PMC12230015; doi:10.3389/fphar.2025.1604711)
Supplement: Supplementary file 1 [file DataSheet1.pdf]

Supplementary Material

Supplementary Table 1. Structural characteristics, ribonuclease and biological activity of miRNA-targeted catalytic nucleic acids (antimiRzymes and miRNases)

Type of catalytic nucleic acids/ structure and chemical modifications	Target	Targeted functional domain of miRNA*	Cleavage efficiency, %	Biological activity in cell cultures and mouse tumor models	Ref.
**10-23 DNAzyme and LNAzymes**<br>Dz - DNA<br>Lz2 or Lz4 - 2 or 4 LNA units<br>*catalytic core*: 15 nt<br>*binding arms*: 7+8 nt<br>Fig. 2A and D	miR-372-5p<br>miR-373-3p	Dz, Lz2 and Lz4: A14U15 (miR-372-5p) and A12U13 (miR-373-3p)	miR-372-5p (1 h, 10h) / miR-373-3p (1 h, 10h)<br>50-fold enzyme excess, 25 mM Mg^{2+}<br>Dz: 7, 28 / 5, 25<br>Lz2: 50, 80 / 27, 73<br>Lz4: 20, 58 / 12, 55<br>Plateau is reached at 10 h	HEK293T, 100 nmol, LF, 36 h:<br>miR-372 expression plasmid and LATS2 UTR reporter (miR-372 mRNA target): restoration of LATS2 level up to 80% (Lz2) and 95% (Lz4)	(Jadhav et al., 2009)
**10-23 DNAzymes and LNAzymes**<br>Dz1 and Dz2 - DNA<br>Lz1 and Lz2 - 2 or 4 LNA units<br>*catalytic core*: 15 nt<br>*binding arms*: 8+9 nt<br>Fig. 2A and D	miR-27a-3p	Dz1 and Lz1: G7U8<br>Dz2 and Lz2: G15U16	10-fold enzyme excess, 25 mM Mg^{2+}, 1 h<br>Dz1 - 35<br>Lz1 - 70<br>Dz2 - 55<br>Lz2 - 75<br>Plateau is reached at 3h	HEK293T, 100 nM, LF, 48 h:<br>25 and 35% decrease in miR-27a by Dz2 and Dz1, respectively; 40 and 50% decrease in miR-27a by Lz2 and Lz1, respectively;<br>10-15% and 25-40 % increase in prohibitin expression (as part of a luciferase vector) by Dz1/Dz2 and Lz1/Lz2, respectively	(Kaur et al., 2010)
**Hammerhead ribozyme,**<br>**10-23 DNAzyme and**<br>**8-17 DNAzyme**<br>hRz and hRz_TLR - RNA<br>Dz1 and Dz2 -DNA<br>hRz and hRz_TLR:<br>*catalytic core*: 22 nt and 22/19 nt, respectively<br>*binding arms*: 6+6 nt;	miR-21-5p<br>pre-miR-21	hRz and hRz_TLR: C9A10<br>Dz1: A10G11<br>Dz2: G18U19	miR-21: 10-fold enzyme excess, 1 h (5 mM Mg^{2+}, 10 mM Mg^{2+}) / pre-miR-21: 50-fold enzyme excess, 15 h (5 mM Mg^{2+}, 10 mM Mg^{2+})<br>hRz: 73, 94 / 3, 5<br>hRz_TLR: 28, 44 / 1, 3	T98G, 100 nM, LF, 24 h:<br>80% and 50 % decrease in miR-21 level by hRz and Dz1, respectively;<br>4.3- and 3.1-fold PTEN upregulation by hRz and Dz1, respectively	(Belter et al., 2016)

Dz1 (8–17 DNAzyme): *catalytic core*: 15 nt,<br>*binding arms*: 8+7 nt<br>Dz2 (10–23 DNAzyme): *catalytic core*: 15 nt,<br>*binding arms*: 7+7 nt<br>Fig. 2A, C and F			| | 25-fold enzyme excess, 15 h<br>Dz1 - 60 - -<br>Dz2 - 15 8 22		
**10-23 DNazyme (conjugated with LDL-R-recognizing aptamer)**<br>Dz1, Dz2, Dz3 and Dz4 - DNA<br>*catalytic core*: 15 nt<br>*binding arms*: Dz1: 14+6, Dz2: 9+6, Dz3: 6+14, Dz4: 6+9<br>Fig. 2A	miR-21	Dz1 and Dz2: A7U8<br>Dz3 and Dz4: A15U16	200-fold miRNA excess, 10 mM Mg^{2+}, 2h<br>Dz1 - 5<br>Dz2 - 0<br>Dz3 - 40<br>Dz4 - 20	Dz3:<br>‣ U87MG, LF, 24 h: 68 and 90% decrease in miR-21 at 100 nM and 200 nM, respectively<br>‣ MDA-MB-231, LF, 24 h: 32, 40 and 54% decrease in miR-21 at 100 nM, 200 nM and 400 nM, respectively<br>‣ U87MG, transferrin receptor aptamer, 24 h: 41 and 52% decrease in miR-21 level at 1 and 2 µM, respectively<br>‣ Huh-7, 2 µM, aptamer delivery, 24 h: 56% decrease in miR-21 level	(Larcher et al., 2019; Wang et al., 2020)
**DNAzymes *de novo* selected**<br>Dz1, Dz2 and Dz3 - DNA<br>*catalytic core*: 15 nt;<br>*binding arms*: Dz1: 15+7, Dz2: 14+8, Dz3: 9+13<br>Fig. 2B	miR-155	Dz1: U8A9<br>Dz2: A9A10<br>Dz3: U14G15	10-fold enzyme excess, 1 mM Zn^{2+}, 12h<br>Dz1 - 75<br>Dz2 - 70<br>Dz3 - 60	no	(Inomata et al., 2021)
**XNazymes *de novo* selected**<br>Dz-17, Dz-20a and Dz-21 – FANA<br>*catalytic core*: Dz-17 and Dz-20a: 17 nt, Dz-21: 18 nt<br>*binding arms*: Dz-17 and Dz-20a: 7+7 nt, Dz-21: 7+6 nt<br>Fig. 2E	miR-17<br>miR-20a<br>miR-21	miR-17 and miR-20a: U9U10<br>miR-21: A10G11	5-fold enzyme excess, 1 mM Mg^{2+}, 8 h<br>Dz-17 - 80<br>Dz-20a - 75<br>Dz-21 - 50<br>vs.<br>8-17 DNazyme - 0<br>10-23 DNazyme - 8<br>hRz_TLR - 30	no	(Donde et al., 2022)

**miRNases (linear)**<br>***peptide and amine-based***<br>*binding domain*: PNA, 14 nt<br>*catalytic domain*:<br>R1: peptide HGG·Cu^{2+};<br>*linker*: PEG at C- and N-terminus<br>R2: Lys-DETA;<br>*linker*: PEG-lysine-succinic and PEG at C- and N-terminus, respectively<br>Fig. 3A (II)	miR-1323	terminal region	Equimolar condition, 1h<br>R1 - 47.5 (2 mM Cu^{2+})<br>R2 - 90<br>Plateau is reached at 1 h	no	(Gaglione et al., 2011)
**miRNases (linear)**<br>***imidazole-based***<br>*binding domain*: PNA, 10 or 15 nt<br>*catalytic domain*: tris(2-aminobenzimidazole<br>*linker* – 6-amino-hexanoic acid, one or two lysine at the C-terminus (for solubility)<br>Fig. 3A (II)	miR-20a	A18G19<br>G19G20	5-fold miRNase excess, 15 h<br>50	no	(Danneberg et al., 2015)
**miRNases (linear and hairpin)**<br>***peptide-based***<br>*binding domains*: DNA<br>R16: 16 nt;<br>H-R14 and H^L-R14: 14 nt with 16 or 22 nt hairpin;<br>H-R16 and H^L-R16: 16 nt with 16 or 22 nt hairpin<br>*catalytic domain*: peptide acetyl-$(LRLRG)_2$<br>*linker*: aminohexyl<br>Fig. 3A (I) and B(I)	miR-21	G15A16, G18U19 and G21A22	20-fold RNase excess<br>— / 8 h / 24 h<br>R16 / 28 / 78<br>H-R14 / 32 / 87<br>H^L-R14 / 25 / 69<br>H-R16 / 9 / 23<br>H^L-R16 / 10 / 18<br>2-fold miRNA excess<br>— / 8 h / 24 h<br>H^L-R14 / 40 / 65	H^L-R14:<br>‣ RLS_{40}, 1 μM, LF:<br>50 % decrease in miR-21 level (24 h),<br>1.7-fold and 2.4-fold increase in PTEN and PDCD4 level (48 h), respectively;<br>50% inhibition of proliferation;<br>‣ B16, 1 μM, LF:<br>induction of apoptosis in 28% of cell population,<br>70% inhibition of invasion<br>‣ **In vivo** model RLS_{40}/CBA/LacSto mice, single in vitro treatment of tumor cells via LF-delivery with subsequent implantation into mice:<br>95% suppression of tumor growth (the effect of corresponding oligonucleotide free of the peptide was 50%)	(Patutina et al., 2017a, 2019)

**miRNases (hairpin)**<br>***peptide-based***<br>*binding domains*: H-R1 and H-R2 – hairpin DNA+2’OMe 14 or 11 nt complementary sequence, respectively, with 16 nt hairpin<br>*catalytic domain*: peptide acetyl-(LRLRG)$_2$<br>*linker*: aminohexyl<br>Fig.3B (II) and (III)	miR-21	G15A16,<br>G18U19 and<br>G21A22	20-fold RNase excess<br>— 8 h / 24 h<br>H-R1: 22 / 63<br>H-R2: 88 / 100<br>2-fold miRNA excess<br>— 8 h / 24 h<br>H-R1: 40 / 41<br>H-R2: 63 / 70	KB-8-5, 0.5 μM, LF, 72 h:<br>58 and 48% inhibition of proliferation by H-R1 and H-R2, respectively; 55% increase in migration activity by H-R2	(Miroshnichenko et al., 2019a)
**miRNases (linear and hairpin)**<br>***peptide-based***<br>*binding domains*: DNA<br>R-21-(DEG)$_2$: 14 nt;<br>H-R-21-(DEG)$_2$: 12 nt with 16 nt hairpin;<br>H^L-R-21-(TrEG)$_2$: 14 nt with 22 nt hairpin;<br>H-R-17-(DEG)$_2$: 14 nt with 16 nt hairpin;<br>H-R-17-DEG: 14 nt with 16 nt hairpin;<br>H-R-17-(DEG)$_2$-AACT: 14 nt with 16 nt hairpin<br>*catalytic domains*: peptide (LR)$_4$G-NH$_2$ or peptide with short oligonucleotide AACT-(LR)$_4$G-NH$_2$<br>*linkers*: DEG, (DEG)$_2$ or (TrEG)$_2$<br>Fig. 3A (I) and B(I)	miR-21<br>miR-17	miR-21:<br>U1A2,<br>C13U14,<br>U14G15,<br>U17G18,<br>U19U20 and<br>U2G21<br><br>miR-17:<br>C17A18 and<br>U21A22	20-fold RNase excess, 24 h<br>— miR-21<br>R-21-(DEG)$_2$: 27<br>H-R-21-(DEG)$_2$: 17<br>H^L-R-21-(TrEG)$_2$: 5<br>— miR-17<br>H-R-17-(DEG)$_2$: 8<br>H-R-17-DEG: 3<br>H-R-17-(DEG)$_2$-AACT: 1.5	H-R-17-(DEG)$_2$:<br>B16, 1 μM, LF:<br>40% and 80% decrease in miR-17 level at 100 nM and 500 nM (24 h);<br>2.2-fold increase in E2F1 (72 h);<br>5-fold inhibition of cell proliferation (120 h)<br>(peptide-free oligonucleotide caused 2.5-fold decrease in cell growth rate)	(Patutina et al., 2018)
**miRNases (dual)**<br>***peptide-based***<br>*binding domain*:<br>DNA with 2-aminoadenine substitutions, 5 nt gap:<br>dual-R-21: 8+9 nt;	miR-21<br>miR-17<br>miR-18a<br>miR-155	Every bond in the gap:<br>central bulge and 3’-supplementary region	20-fold RNase excess, 8 mM Mg^{2+}, 24 h<br>dual-R-21 - 30<br>dual-R-17 - 18<br>dual-R-18a - 18<br>dual-R-155 - 55	no	(Patutina et al., 2020b)

dual-R-17: 8+10 nt;<br>dual-R-18a: 7+10 nt;<br>dual-R-155: 8+10 nt<br>*catalytic domain*: peptide acetyl-$(LRLRG)_2$<br>*linkers*: aminohexyl and thiohexyl<br>Fig. 3C					
**miRNases (bulge-forming - BF)**<br>***peptide-based***<br>*binding domains* DNA (bulge-forming, 3 nt bulge)<br>mono-BF-R-21-α1, mono-BF-R-21-α2, mono-BF-R-21-β1 and mono-BF-R-21-β1 (mono-peptide): 19 nt;<br>mono-BF-R-17-α, mono-BF-R-17-β (mono-peptide), bi-BF-R-17-αα and bi-BF-R-17-ββ (bi-peptide): 20 nt<br>*catalytic domain*: one or two peptides acetyl-$(LRLRG)_2$<br>*linkers:* aminohexyl linkers attached at the C1’ position of abasic sugar residues in α (dRα) or β (dRβ) configuration<br>Fig. 3D	miR-21<br>miR-17	Every bond in the bulge-loop:<br>central bulge	20-fold RNase excess, 8 mM Mg^{2+}, 24 h<br>**miR-21**<br>mono-BF-R-21-α1: 4<br>mono-BF-R-21-α2: 2<br>mono-BF-R-21-β1: 9<br>mono-BF-R-21-β1: 4<br>**miR-17**<br>mono-BF-R-17-α: 17<br>mono-BF-R-17-β: 9<br>bi-BF-R-17-αα: 32<br>bi-BF-R-17-ββ: 65	no	(Patutina et al., 2022)
**miRNases (crab-like)**<br>***peptide-based***<br>*binding domains*: DNA 13 nt<br>*catalytic domain*: one or two peptides acetyl-$(LRLRG)_2$<br>*linkers:* 5’ aminohexyl and 3’ 6-amino-2(hydroxymethyl)hexyl or 5’ and 3’ aminohexyl linkers attached at the C1’ position of abasic sugar residues in α (dRα) or β (dRβ) configuration<br>Fig. 3 E	miR-21<br>miR-17	miR-21:<br>U1A2, G3C4, C4U5, U5U6, U6A7, C9A10<br>miR-17:<br>C1A2, U6G7, C17A18, U21U22	**miR-21**<br>(20-fold RNase excess, 24 h / 2-fold miRNA excess, 8 h / 2-fold miRNA excess, 24 h)<br>3’-mono-pep-R-21: 15 / - / -<br>5’-mono-pep-R-21: 7 / - / -<br>crab-R-21: 84 / 50 / 99<br>crab-R-21-α: 32 / 6 / 25<br>crab-R-21-β: 64 / 13 / 37	crab-R-21 and crab-R-17-α:<br>‣ MCF7, 1 μM, LF:<br>65% (peptide-free oligonucleotide - 30%) and 58% (peptide-free oligonucleotide - 40%) decrease in miR-21 and miR-17 levels by crab-R-21 and crab-R-17-α, respectively (24 h);<br>1.7-fold and 1.9-fold increase in PDCD4 and E2F1 by crab-R-21 and crab-R-17-α, respectively (72 h);<br>50% inhibition of proliferation by crab-R-21 and crab-R-17-α (72 h)<br>‣ A549, 1 μM, LF:<br>80% inhibition of proliferation by crab-R-21 (72h)	(Chiglintseva et al., 2024)

<table>
<tr>
<td></td>
<td></td>
<td></td>
<td>
<table>
<tr><td colspan="4">miR-17</td></tr>
<tr><td colspan="2">20-fold RNase excess</td><td colspan="2">2-fold miRNA excess</td></tr>
<tr><td></td><td>24 h</td><td>8 h</td><td>24 h</td></tr>
<tr><td>crab-R-17-α</td><td>100</td><td>67</td><td>85</td></tr>
<tr><td colspan="4">Plateau is reached at 36 h (100 % cleavage) for miR-21 and at 12 h for miR-17 (100% cleavage)</td></tr>
</table>
</td>
<td>‣ In vivo xenograft model MCF7/nude mice, single in vitro treatment of tumor cells via LF-delivery with subsequent implantation into mice: 85% suppression of tumor growth; 3-fold increase in mitotic and proliferative activity, 1.5-fold increase in the number of apoptotic cells in tumor tissue</td>
<td></td>
</tr>
</table>

* The color of a site reflects its belonging to a specific functional domain in the miRNA sequence: pink – seed region, blue – central bulge, orange – 3’ supplementary region, and purple – terminal region

LNA – locked nucleic acids; FANA – 2’-deoxy-2’-fluoro-β-D-arabino nucleic acid; PNA – peptide nucleic acids; LDL-R – low-density lipoprotein receptor; DETA – diethylenetriamine; DEG – diethylene glycol; TrEG - tetraethylene glycol; LF - Lipofectamine delivery
